# Porous sheet-like carbon nitride with high porosity for efficient visible-light-driven hydrogen evolution reaction

**DOI:** 10.1039/d6ra05550h

**Published:** 2026-09-01

**Authors:** Zhidong Yang, Xiaodong Deng, Jinjia Wang, Peixia Li

**Affiliations:** a School of Chemistry and Environment, Ankang University Ankang 725000 Shanxi China yangzd1618@163.com l485276@163.com

## Abstract

Carbon nitride (CN) has emerged as a promising metal-free photocatalyst; however, its practical application remains severely hindered by insufficient active sites and rapid charge recombination. Herein, we report a facile three-step synthetic strategy to construct porous sheet-like CN (PSCN) with high porosity. Specifically, urea and melamine were dissolved in hot water (100 °C) and evaporated to dryness under stirring to afford a homogeneous precursor, which was subsequently calcined in a muffle furnace to form tubular CN (TCN). A further thermal treatment in air using a tube furnace then triggered the structural transformation from the tubular to porous sheet-like morphology, thereby endowing the catalyst with an enlarged specific surface area, abundant porous channels, and efficient charge separation. Benefiting from these structural merits, the as-obtained PSCN exhibits significantly enhanced photocatalytic hydrogen evolution activity under visible light irradiation (*λ* ≥ 420 nm), achieving a H_2_ production rate of 1278 µmol g^−1^ h^−1^, which is about 5 times higher than that of TCN. This work offers a simple and scalable approach to tailor the morphology and electronic structure of CN, thus holding great promise for high-performance photocatalytic energy conversion.

## Abstract 2

High-porosity carbon nitride sheets are fabricated *via* a facile three-step strategy. The porous 2D architecture offers abundant active sites and fast charge separation toward efficient visible-light photocatalytic H_2_ evolution.

## Graphical abstract

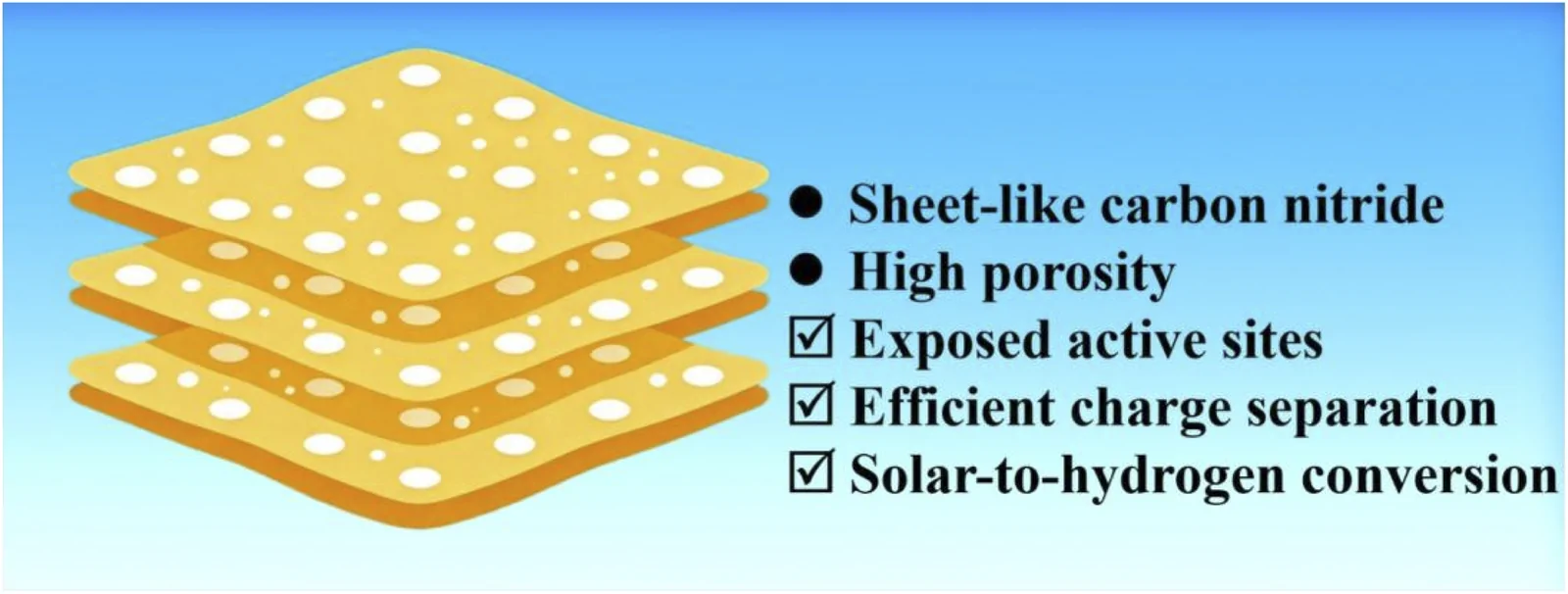

## 1. Introduction

Photocatalytic water splitting for sustainable hydrogen production has emerged as a viable strategy to mitigate the dual challenges of global energy scarcity and environmental degradation, enabling the conversion of renewable solar energy into storable chemical energy.^1–3^ Metal-free carbon nitride (CN) emerges as a highly promising candidate in the field of photocatalysis,^4–6^ which has attracted considerable research attention owing to its chemical stability, band structure aligned for visible-light absorption, scalable synthesis derived from low-cost nitrogen-rich precursors, and intrinsic non-toxicity.^7–9^ However, the photocatalytic performance of pristine CN is severely constrained by inherent drawbacks, including insufficient specific surface area, and rapid recombination of photogenerated electron–hole pairs, which hinder its large-scale industrial implementation.^10–12^ To overcome these bottlenecks, extensive efforts have been devoted to tailoring the electronic structure, crystal plane orientation, and microarchitecture of CN, among which morphological engineering has been proven to be one of the most effective strategies to optimize its photocatalytic behaviour.

In recent years, the design and synthesis of one-dimensional (1D) tubular carbon nitride (TCN) have attracted significant research interest.^13,14^ The tubular architecture promotes the transport of photogenerated charge carriers, thereby conferring superior photocatalytic performance compared to bulk carbon nitride materials.^15–17^ Nevertheless, TCN still suffers from limited exposed active sites and insufficient pore structure, which restricts the full contact between the catalyst surface and reactant molecules, as well as the efficient separation of photogenerated charges.^18–21^ Further structural optimization to transform 1D TCN into two-dimensional (2D) porous sheet-like architectures is highly desirable, as 2D porous nanosheets feature ultra-thin thickness and abundant porous channels, which can simultaneously expose more edge active sites, facilitate mass transfer, and charge separation.^22–25^ While initial proof-of-concept studies have demonstrated the feasibility of this transformation, most existing routes still require complex auxiliary processes, harsh reaction conditions, or additional post-synthetic modification, and frequently produce materials with relatively low porosity that limits further improvement of photocatalytic activity. Therefore, the development of a facile, low-cost strategy to construct 2D porous sheet-like CN with high porosity for efficient visible-light-driven hydrogen evolution reaction (HER) remains a critical unmet challenge.

**Fig. 1:**
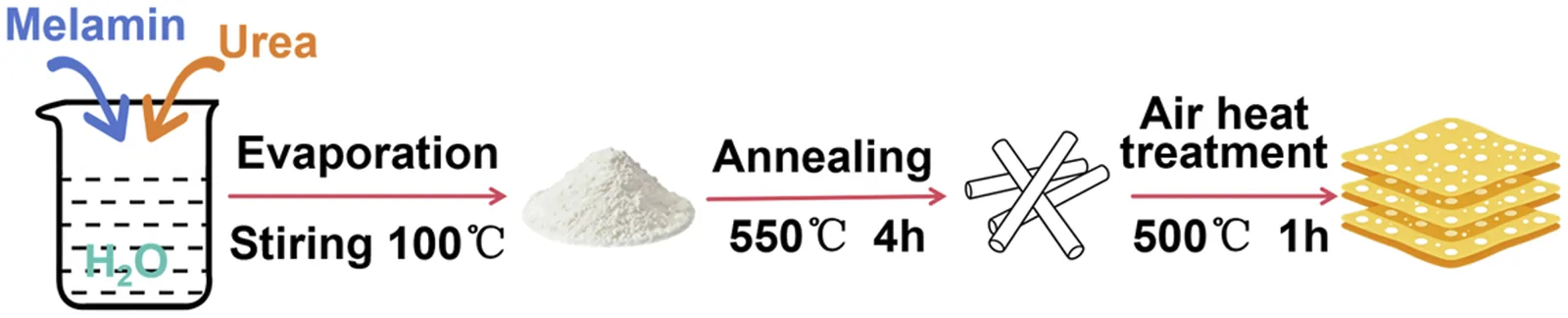
Schematic illustration of the processes for synthesizing TCN and PSCN.

Herein, we report a facile three-step synthesis of PSCN: urea and melamine are dissolved in hot water (100 °C), followed by constant-temperature evaporation to obtain a uniform precursor, which is then calcined in a muffle furnace to form TCN, and subsequently thermally treated in air using a tube furnace to transform into PSCN as shown in Fig. 1. This strategy avoids any harsh reagents, exotic templates or complicated purification steps, and delivers PSCN with much higher porosity. The high porosity of PSCN endows it with abundant surface active sites, accelerated charge separation and mass transfer, resulting in a significantly improved visible-light-driven HER performance compared to TCN and many reported CN-based photocatalysts. This work provides a facile and scalable route to high-performance 2D porous carbon nitride photocatalysts, opening new opportunities for practical solar-to-fuel conversion.

## 2. Experimental section

### 2.1 Chemicals

All chemicals were analytical-grade and used without further purification. Melamine (≥99.0%), urea (≥99.0%) and triethanolamine (≥98%) were purchased from Sinopharm Chemical Reagent Co., Ltd.

### 2.2 Preparation of a urea–melamine mixture

Firstly, melamine (9.8 g) and urea (0.2 g) were added to 200 mL of ultrapure water. The mixture was homogenized and then gradually heated to 100 °C to give a transparent solution. Subsequently, the solution was maintained at 100 °C until complete evaporation. Finally, the resulting white powder was dried under vacuum at 40 °C overnight to afford the urea–melamine mixture.

### 2.3 Synthesis of TCN

TCN was prepared using urea–melamine mixture as precursor by thermal polymerization. Typically, 10 g mixture was calcined at 550 °C for 4 h in muffle furnace (heating rate: 5 °C min^−1^). And then, when the temperature was lower than 30 °C, the yellow powder was collected and denoted as TCN.

### 2.4 Synthesis of PSCN

First, 0.2 g of TCN was placed in a porcelain boat, which was then positioned at the centre of a tube furnace. The furnace was heated to 500 °C at a ramp rate of 5 °C min^−1^ under an air atmosphere, and this temperature was maintained for 1 h. Subsequently, the system was cooled to room temperature, and the resulting sample was collected and denoted as PSCN.

### 2.5 Characterization

The morphology was observed by scanning electron microscope (SEM, SU8010, Japan). The crystalline structures were studied by an X-ray diffractometer (XRD, D8, Germany). The N_2_ adsorption–desorption isotherms were obtained on a fully automatic adsorption instrument (BET, Micromeritics ASAP 2460, America). The functional groups were measured on a Fourier transform infrared spectrometer (FT-IR, FTIR200, Germany). The chemical compositions were determined by an X-ray photoelectron spectrometer (XPS, Thermo Scientific K-Alpha, America). The UV-vis spectra of the photocatalysts powder were obtained on a UV-visible spectrophotometer (UH4150, Japan). The photoluminescence was recorded by using Edinburgh spectrofluorometer (PL, FLS1000, the United Kingdom), and the excitation wavelength was 365 nm.

The electrochemical measurements were performed on an electrochemical workstation (CHI760C, Chinstruments, China) using a three-electrode system. The Pt sheet and Ag/AgCl electrode were used as the counter electrode and reference electrode, respectively. The electrolyte was Na_2_SO_4_ solution (0.2 M). The working electrode was prepared by dip-coating catalyst slurry on FTO conductive glass (1 × 1 cm) and then dried in the air.

### 2.6 Photocatalytic hydrogen evolution test

The photocatalytic hydrogen production experiments were performed on an online photocatalytic hydrogen production system (Labsolar 6A, Beijing Perfectlight) at ambient temperature. A 300 W Xenon arc lamp with a cutoff filter (*λ* > 420 nm) was employed as the visible light source, and the light intensity was calibrated to 100 mW cm^−2^ to trigger the photocatalytic reaction. Typically, 20 mg of the catalysts were added in aqueous triethanolamine solution (50 mL, 10 vol%) with ultrasonic dispersion for 5 min. Then appropriate amount H_2_PtCl_6_ was added to make sure the photocatalysts loaded with 3wt% Pt by photodeposition method. Prior to the reaction, the mixture was degassed under vacuum, and then filled with high-pure Ar (99.99%, 1 atm). The reaction temperature was maintained at 30 °C by a constant temperature cooling circulation pump. After every one hour, the generated hydrogen gas was measured on an online gas chromatograph (GC9790II PLF-01) with thermal conductivity detector (TCD) using N_2_ as the carrier gas. For the cyclic stability test, after each photocatalytic stability test cycle, the used catalyst was collected by centrifugation at 8000 rpm for 10 min, washed repeatedly with ethanol and deionized water to remove residual sacrificial agent, and dried under vacuum at 60 °C overnight for the next round of reaction.

## 3. Results and discussion

**Fig. 2:**
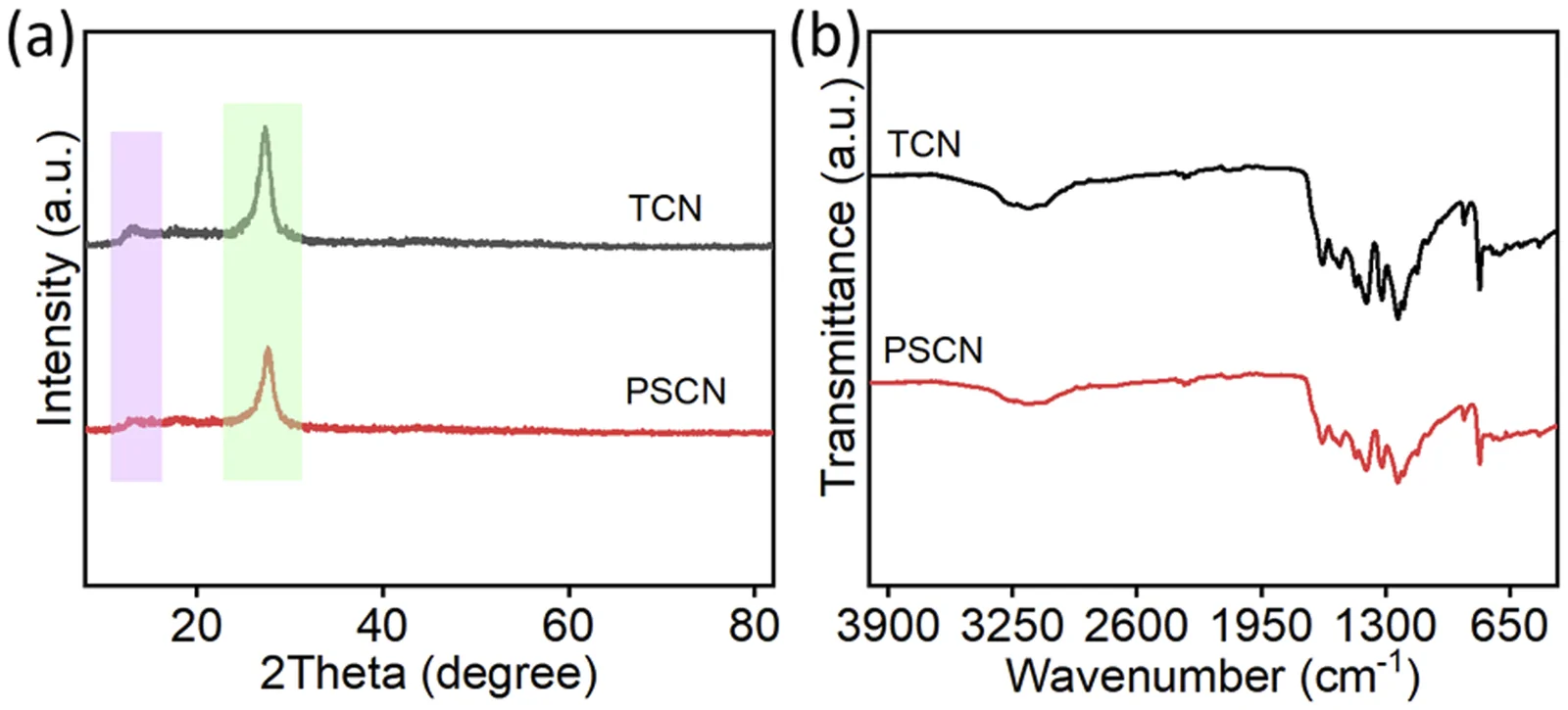
(a) XRD patterns of TCN and PSCN, (b) FTIR spectra of TCN and PSCN.

X-ray diffraction (XRD) was employed to characterize the crystal structures of TCN and PSCN. As shown in Fig. 2a, TCN exhibits two characteristic diffraction peaks at 13.0° and 27.5°, which are assigned to the (100) plane (corresponding to the in-plane repeating units of the continuous heptazine framework) and the (002) plane (arising from the stacking of conjugated aromatic structures along the *c*-axis), respectively.^26^ PSCN displays an XRD pattern similar to that of TCN, indicating that the air-thermal treatment does not alter the intrinsic crystal structure of TCN. Notably, the (001) diffraction peak of PSCN is slightly attenuated, while the (002) peak exhibits both reduced intensity and a shift to higher angles (Fig. S1). The attenuated (001) peak suggests the disruption of in-plane long-range order of tri-*s*-triazine units, whereas the altered (002) peak indicates reduced interlayer stacking order and a decreased interlayer distance, as deduced from Bragg's law.^27,28^ The surface properties of all samples were further investigated by Fourier transform infrared spectroscopy (FTIR). As revealed in Fig. 2b, the peak at 810 cm^−1^ originates from the characteristic breathing mode of triazine ring.^29^ The multiple bands in the region of 1200 to 1700 cm^−1^ are attributed to the typical vibration modes of C–N heterocycle.^30–32^ The broad peak at 3000–3500 cm^−1^ can be ascribed to the stretching vibrations of N–H and/or O–H bonds.^33^ Noticeably, no obvious changes can be observed, suggesting that the basic structure of TCN after morphology transformation can be well maintained.

**Fig. 3:**
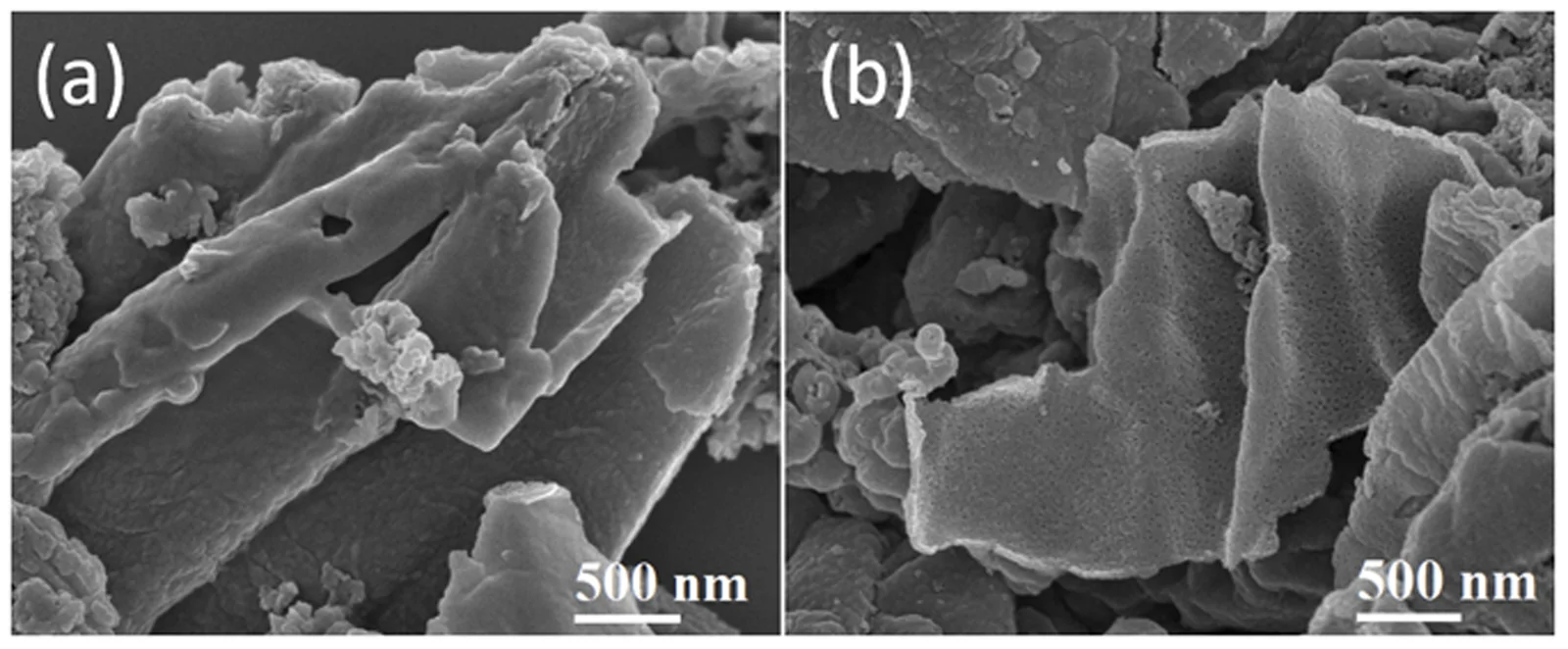
(a) SEM image of TCN, (b) SEM image of PSCN.

The catalytic activity of carbon nitride (CN) is strongly dependent on its morphological features. Typically, 2D or thin-layered CN architectures generally outperform their bulk counterparts, owing to their increased exposed active sites and improved dispersibility in solution.^25^ Scanning Electron Microscopy (SEM) were used to confirm the morphology of the prepared samples. As depicted in Fig. 3a, the sample prepared after primary calcination in the muffle furnace shows a well-defined tubular structure with a smooth surface. In striking contrast, after the secondary thermal treatment in the tubular furnace, the as-obtained product (Fig. 3b) undergoes a distinct morphological transformation from 1D tubular structure to 2D nanosheet architecture. Notably, the obtained nanosheets are highly porous with uniformly distributed pores throughout the entire structure (Fig. S2), endowing the sample with significantly enhanced porosity. Based on the observed morphological and structural evolution from the primary calcined product TCN to the final PSCN, we infer that the morphological transformation during secondary calcination is most likely induced by the synergistic effect of structural relaxation, partial decomposition of the incompletely polycondensed carbon nitride framework, and *in situ* release of gaseous products generated during the air thermal treatment process. This process not only triggers the collapse and reconstruction of the tubular precursor but also generates abundant hierarchical pores within the final sheet-like carbon nitride. Systematic *in situ* experimental investigations are still required to further validate this transformation process in future work.

**Fig. 4:**
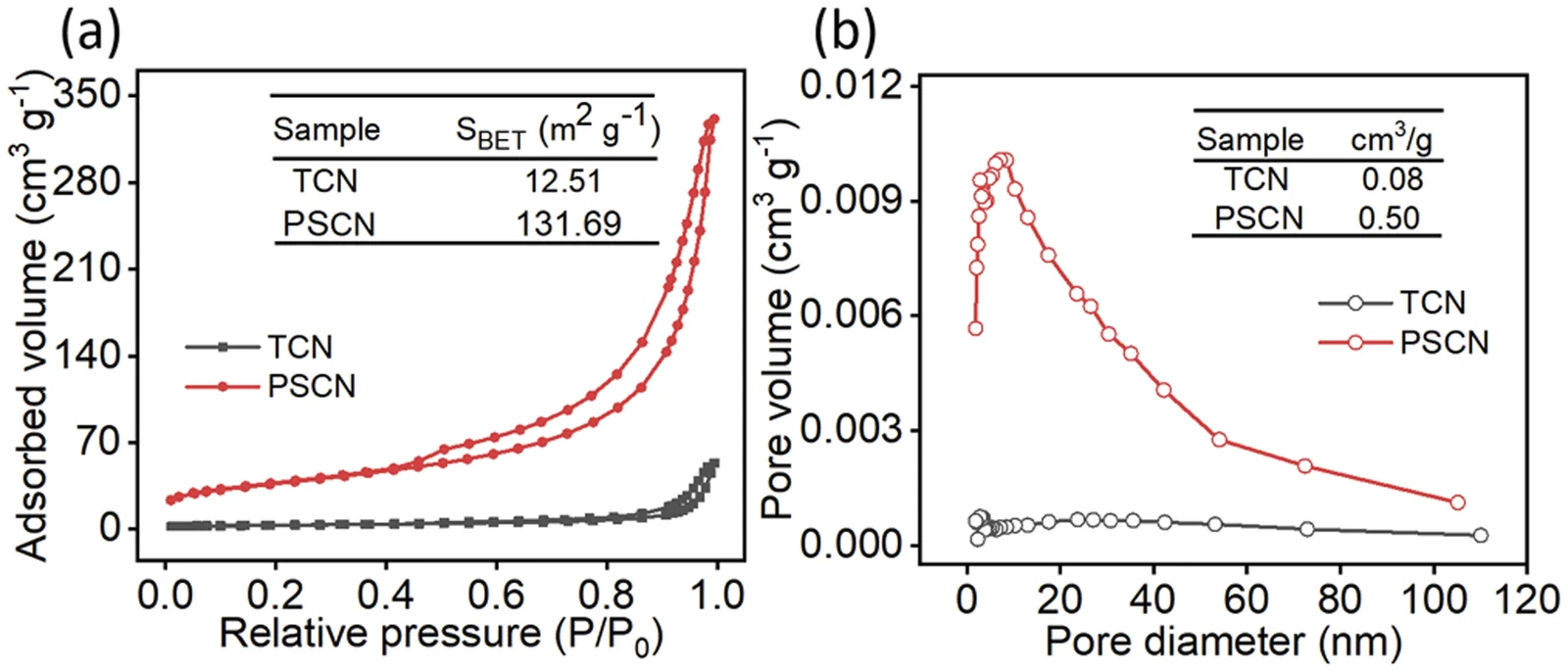
(a) N_2_ adsorption–desorption isotherm curves, (b) pore size distribution curves of TCN and PSCN sample.

The specific surface area, pore size distribution, and pore volume of the as-prepared samples were tested using N_2_ adsorption–desorption isotherms. As shown in Fig. 4a and S3, all samples exhibit type IV sorption isotherms with H_3_ hysteresis loops, indicating the presence of mesopores in the material.^34^ The Brunauer–Emmett–Teller (BET) specific surface area of the PSCN is calculated to be 131.69 m^2^ g^−1^ with a nearly 10-fold increasement relative to the TCN (12.51 m^2^ g^−1^). A dramatic increase in specific surface area and pore volume demonstrates that PSCN probably relates to the increased voids/cavities. Pore size distribution analysis further quantifies the substantial difference in pore structure between TCN and PSCN (Fig. 4b and S4). TCN exhibits extremely low pore volume across the entire measured pore size range, with a total pore volume of only 0.08 cm^3^ g^−1^, indicating a dense bulk structure with negligible mesopores or macropores. In sharp contrast, PSCN displays a distinct pore size distribution with a prominent broad peak centered at 2–20 nm, demonstrating the generation of abundant uniform mesopores, alongside a continuous pore volume distribution in the macropore region (>20 nm). This confirms the formation of a hierarchical interconnected porous structure.^35^ Notably, the total pore volume of PSCN reaches 0.50 cm^3^ g^−1^, which is over 6 times higher than that of TCN, verifying the ultrahigh porosity of PSCN. These results demonstrate that the modification process successfully constructs a well-developed porous structure in PSCN with ultrahigh porosity, which is highly favorable for exposing active sites and accelerating mass transfer during photocatalysis.^36^

**Fig. 5:**
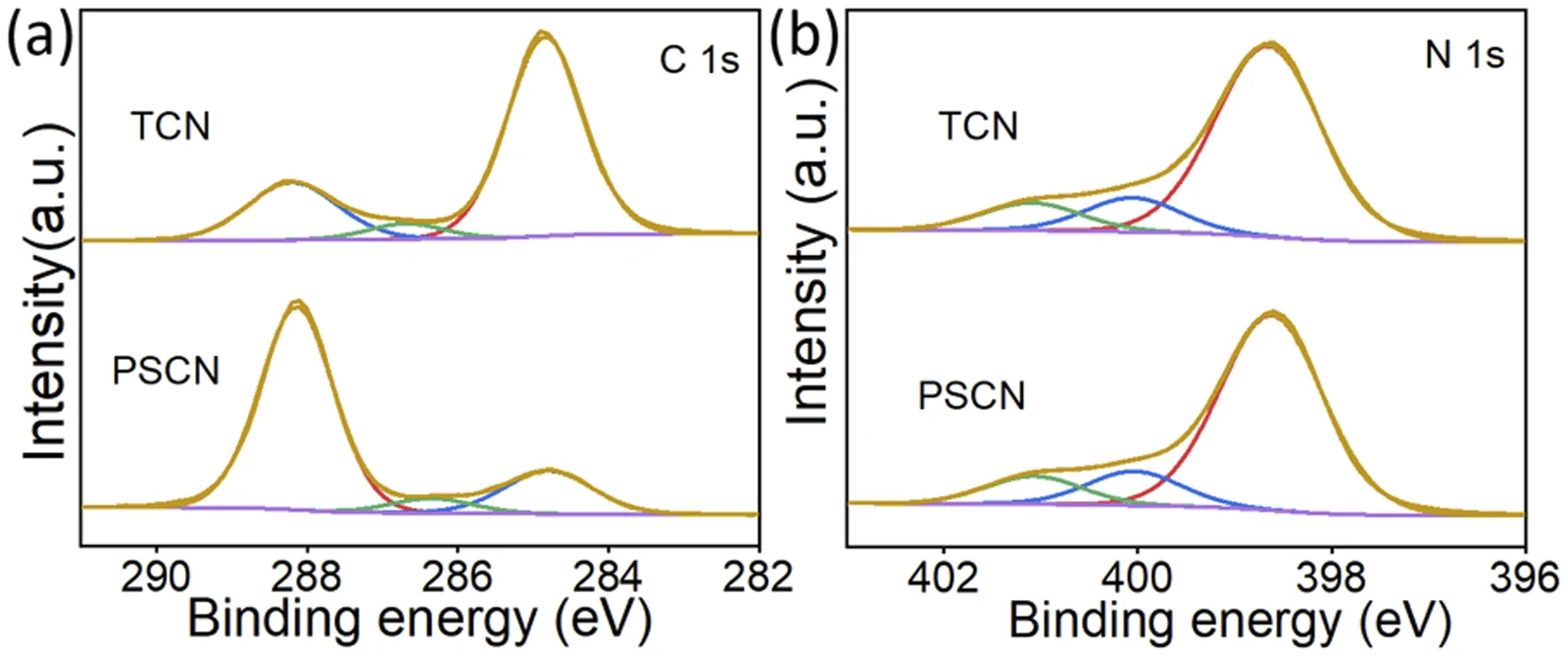
High-resolution (a) C 1s and (b) N 1s spectra of TCN and PSCN sample.

X-ray photoelectron spectroscopy (XPS) was employed to probe the chemical states and surface elemental compositions of the prepared samples. The survey XPS spectra of TCN and PSCN (Fig. S5 and S6) confirm the presence of C, N, and O elements, with weak O 1s signals ascribed to adventitious surface-adsorbed oxygen-containing species.^26^ The high-resolution C 1s XPS spectra of TCN and PSCN (Fig. 5a) can be deconvoluted into three distinct peaks at ∼288.1, ∼286.3, and ∼284.8 eV, assigned to sp^2^-hybridized carbon in N–C=N moieties, C–NH_*x*_ groups at the edges of heptazine units, and C–C/C=C bonds from graphitic carbon impurities, respectively.^37–39^ Analogously, the N 1s spectra of both samples (Fig. 5b) exhibit three deconvoluted peaks at ∼401.1, ∼400.0, and ∼398.6 eV, corresponding to terminal amino groups (C–NH_2_), tertiary nitrogen (N–(C)_3_), and sp^2^-hybridized N atoms in tri-*s*-triazine rings (C–N=C), respectively.^24,37^ Quantitative XPS analysis further reveals that the relative contents of C and N species in different chemical environments (Table S1) are comparable between TCN and PSCN, with negligible binding energy shifts (<0.05 eV) of the characteristic peaks. These data indicate that the intrinsic chemical bonding environment of CN does not change significantly during the morphological transformation from bulk TCN to the PSCN. Given that our morphological regulation strategy only tailors the stacking mode and dimensional architecture of carbon nitride without disrupting its fundamental tri-*s*-triazine conjugated skeleton, it is reasonable to infer that the core electronic structure of the conjugated backbone is largely unaltered, with the π-electron delocalization of the conjugated system and the relative content of terminal functional groups retained at a stable level. The consistency in intrinsic electronic structure suggests that the potential catalytic performance difference between TCN and PSCN is more likely ascribed to morphological regulation-induced factors, including increased specific surface area, more exposed active sites, and optimized mass transfer efficiency.

**Fig. 6:**
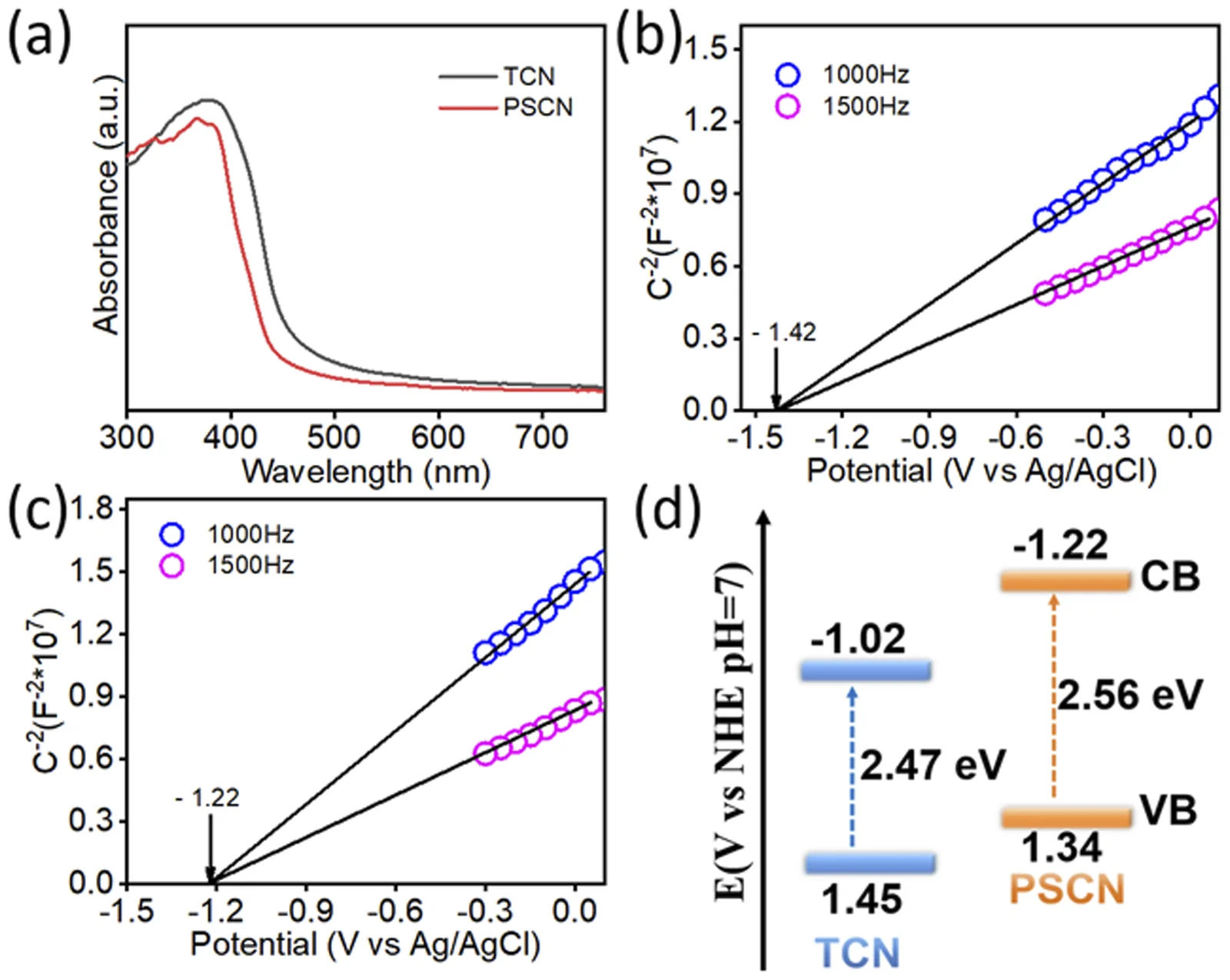
(a) UV-vis absorption spectra, (b and c) Mott–Schottky plots collected at various frequencies *versus* the saturated Ag/AgCl reference electrode (pH = 7), (d) band structure alignments of TCN and PSCN, respectively.

Optical properties of the as-prepared catalysts were investigated *via* UV-vis absorption spectroscopy (Fig. 6a). TCN exhibits intrinsic absorption spanning the UV to visible region, with an absorption edge at ∼470 nm. Obviously, the absorption edge of PSCN displays a blue shift with respect to TCN, originating from the quantum size effect of the thinner thickness. The energy band gap (*E*_g_) of the samples were estimated based on the UV-vis absorption spectra using the Tauc model.^33^ According to the Kubelka–Munk function, the bandgaps are 2.47 eV for the TCN and 2.56 eV for the PSCN, respectively (Fig. S7). To ensure that the prepared catalysts are suitable for photocatalytic hydrogen production under visible light, Mott–Schottky plots of TCN and PSCN at the different frequencies of 1.0, 1.5 kHz (Fig. 6b and c) are analyzed to determine the electronic band structure.^40,41^ The positive slopes of TCN and PSCN are derived from typical n-type characteristic semiconductors.^42^ The flat band potentials of the TCN and the PSCN are determined to be −1.22 and −1.42 V *versus* Ag/AgCl, corresponding to −1.02 and −1.22 *versus* the normal hydrogen electrode (NHE), respectively. Since it is generally accepted that the conduction band (CB) potential in n-type semiconductors is approximately equal to the flat band potential, thus the CB edges of TCN and PSCN are determined to be −1.02 and −1.22 V, respectively. The corresponding band structure alignments of TCN and PSCN are depicted in Fig. 6d. Compared with the TCN, a negative shift the potential of CB was occurred in the PSCN, indicating a slightly enhanced thermodynamic driving force for photocatalytic hydrogen production.^43–45^

**Fig. 7:**
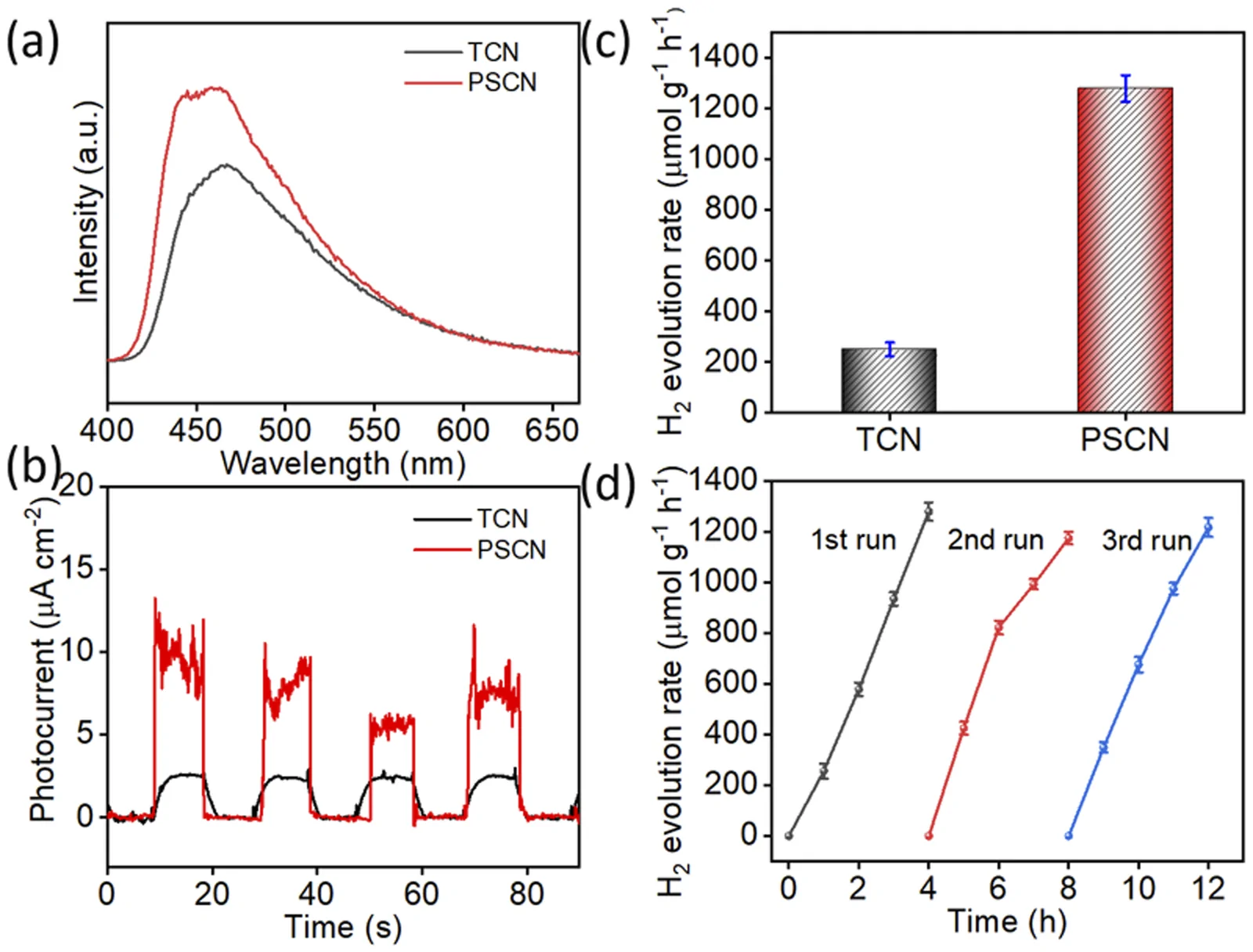
(a) Steady-state PL spectra, (b) transient state photocurrent spectra of TCN and PSCN, (c) photocatalytic H_2_ evolution rate of TCN and PSCN, (d) photocatalytic H_2_ stability test of the PSCN.

Steady-state photoluminescence (PL) emission spectroscopy was employed to further probe the charge separation and transfer kinetics.^46^ As shown in Fig. 7a, the PL intensity of PSCN is lower than that of TCN, suggesting more efficient separation and transfer of photogenerated charge carriers. These PL results confirm that the sheet-like high-porosity morphological modification alleviates charge recombination and extends the charge carrier lifetime of PCN, thereby enhancing the probability of charge carriers participating in photo-redox reactions. Notably, the red-shifted maximum PL emission peak of PSCN relative to TCN is consistent with its enlarged band gap. To further characterize charge carrier behavior, transient photocurrent response (TPR) measurements were performed on TCN and PSCN using a conventional three-electrode cell under intermittent visible-light irradiation. Fig. 7b displays the transient photocurrent curves over four on–off cycles, revealing that PSCN exhibits a significantly higher current density than TCN, indicative of enhanced electron–hole separation efficiency.^39^ Furthermore, electrochemical impedance spectroscopy (EIS) Nyquist plots were employed to evaluate the migration dynamics of photoexcited carriers. As shown in Fig. S8, the Nyquist plot clearly reveals that PSCN has a smaller semicircular arc diameter than TCN. Since the semicircular arc diameter corresponds to charge transfer resistance, the reduced diameter of PSCN indicates lower charge transfer resistance and enhanced electronic conductivity,^47^ consistent with the PL and TPR results.

Photocatalytic H_2_ evolution activities of TCN and PSCN were evaluated under visible-light irradiation (*λ* > 420 nm) with 3 wt% Pt as the cocatalyst. Triethanolamine (TEOA) was selected as the sacrificial reagent owing to its appropriate redox potential, capacity to generate an alkaline environment, and excellent hydrophilicity, which enables effective adsorption onto the surface of CN.^48^ Time-dependent H_2_ production curves over the as-prepared photocatalysts within 4 h were depicted (Fig. S9). It was found that the photocatalytic hydrogen production activity significantly increased upon morphological transformation. The average H_2_ evolution rates (Fig. 7c) further confirm this improvement. The PSCN hydrogen production rate is up to 1278 µmol g^−1^ h^−1^, about five times as high as TCN (250 µmol g^−1^ h^−1^). The inferior activity of TCN is attributed to rapid charge carrier recombination and low specific surface area. The enhanced activity of PSCN is presumably ascribed to its larger specific surface area and porous sheet-like architecture, which facilitate photogenerated charge carrier transport. Notably, the photocatalytic performance of PSCN outperforms previously reported morphology-modified CN-based photocatalysts (Table S2). Stability is another critical criterion for practical photocatalytic applications. The long-term durability of the as-prepared PSCN was further evaluated for practical visible-light-driven hydrogen evolution. As shown in Fig. 7d, no discernible decrease in the H_2_ production rate was observed after three consecutive cycling tests, indicating the outstanding activity stability of the PSCN during photocatalytic reaction. To confirm the structural and chemical stability of PSCN after photocatalytic reaction, post-reaction XRD and FTIR characterizations of the used catalyst were conducted, and the corresponding results are provided in Fig. S10 and S11. No obvious changes in diffraction peak positions/intensities (XRD) or characteristic functional group absorption bands (FTIR) were detected between fresh and used PSCN, demonstrating that the hierarchical porous sheet-like structure and chemical constitution of PSCN are well preserved after photocatalytic reaction.

Porous sheet-like carbon nitride with high porosity enables efficient visible-light-driven hydrogen evolution reaction *via* the synergistic interplay of three key features. First, the large specific surface area originating from the porous sheet-like architecture offers abundant accessible active sites. This facilitates the adsorption and diffusion of reactants (*e.g.*, H_2_O molecules and H^+^) onto the catalyst surface, thus enhancing the probability of surface catalytic reactions. Second, the interconnected porous channels act as highly efficient transmission pathways, which synergistically shorten two key diffusion distances: that of photoexcited electrons migrating from the interior of the nanosheets to their surface, and that of reactants permeating from the surface to the interior along the hierarchical porous framework. These structure-induced advantageous characteristics exert dual pivotal effects: on one hand, by minimizing the migration distance of charge carriers, they significantly reduce the bulk recombination probability of photogenerated electrons and holes, thus retaining a larger quantity of active charge species that can participate in redox reactions; on the other hand, they effectively eliminate the diffusion limitation of reactants and products, substantially enhancing mass transport efficiency to ensure a continuous supply of reactants to active sites and the prompt removal of reaction byproducts. Furthermore, the sheet-like configuration with optimized interlayer spacing provides an extra structural advantage: it offers unobstructed pathways for the separation and rapid migration of photogenerated charge carriers, thereby efficiently suppressing the radiative recombination of electron–hole pairs and leading to a notable improvement in charge separation efficiency, which further guarantees that more photogenerated electrons migrate to the catalyst surface to participate in the reduction reaction. Third, the material possesses an enhanced thermodynamic driving force for reduction, as its conduction band potential is sufficiently negative to thermodynamically favor the reduction of H^+^ to H_2_ under visible light irradiation. In short, the large specific surface area enhances reactant accessibility, efficient charge separation ensures effective electron utilization, and strong thermodynamic driving force promotes the reduction reaction, synergistically boosting the visible-light-driven hydrogen evolution performance.

## 4. Conclusions

In summary, we have developed a facile three-step strategy to transform tubular CN into porous sheet-like architectures, addressing the critical limitations of insufficient active sites and inefficient charge separation in 1D tubular counterparts. Systematic characterizations confirm that the air thermal treatment modulates the crystal structure (reduced interlayer stacking distance, weakened van der Waals interactions) and photoelectric properties (blue-shifted absorption edge, enhanced charge separation kinetics) while preserving the intrinsic carbon-nitrogen coordination environment. Benefiting from the unique porous sheet-like structure with abundant exposed active sites and optimized charge separation, the resulting PSCN photocatalyst exhibits excellent H_2_ evolution activity and stability under visible light irradiation. This work provides an important contribution for inspiring the rational design of high-performance metal-free photocatalysts for solar energy conversion.

## Author contributions

Zhidong Yang: conceptualization, methodology, formal analysis, visualization, validation, writing – original draft. Xiaodong Deng: investigation, data curation. Jinjia Wang: data curation, validation. Peixia Li: supervision, project administration, funding acquisition, writing – review & editing.

## Conflicts of interest

There are no conflicts to declare.

## Supplementary Material

RA-OLF-D6RA05550H-s001

## Data Availability

The data supporting this article have been included as part of the supplementary information (SI). Supplementary information is available. See DOI: https://doi.org/10.1039/d6ra05550h.
