# Supplementary material for: Porous sheet-like carbon nitride with high porosity for efficient visible-light-driven hydrogen evolution reaction
Source: RSC Adv. 2026 Sep 1;16(42):51451–8. doi: 10.1039/d6ra05550h (PMC13531424; doi:10.1039/d6ra05550h)
Supplement: Supplementary file 1 [file RA-016-D6RA05550H-s001.pdf]

Supplementary Information (SI) for RSC Advances.
This journal is © The Royal Society of Chemistry 2026

Supporting Information

Porous sheet-like carbon nitride with high porosity for efficient visible-light-driven hydrogen evolution reaction

Zhidong Yang,* Xiaodong Deng, Jinjia Wang, Peixia Li *

School of chemistry and Environment, Ankang University, Ankang 725000, Shanxi, China.

*Corresponding authors:

E-mail: yangzd1618@163.com; l485276@163.com.

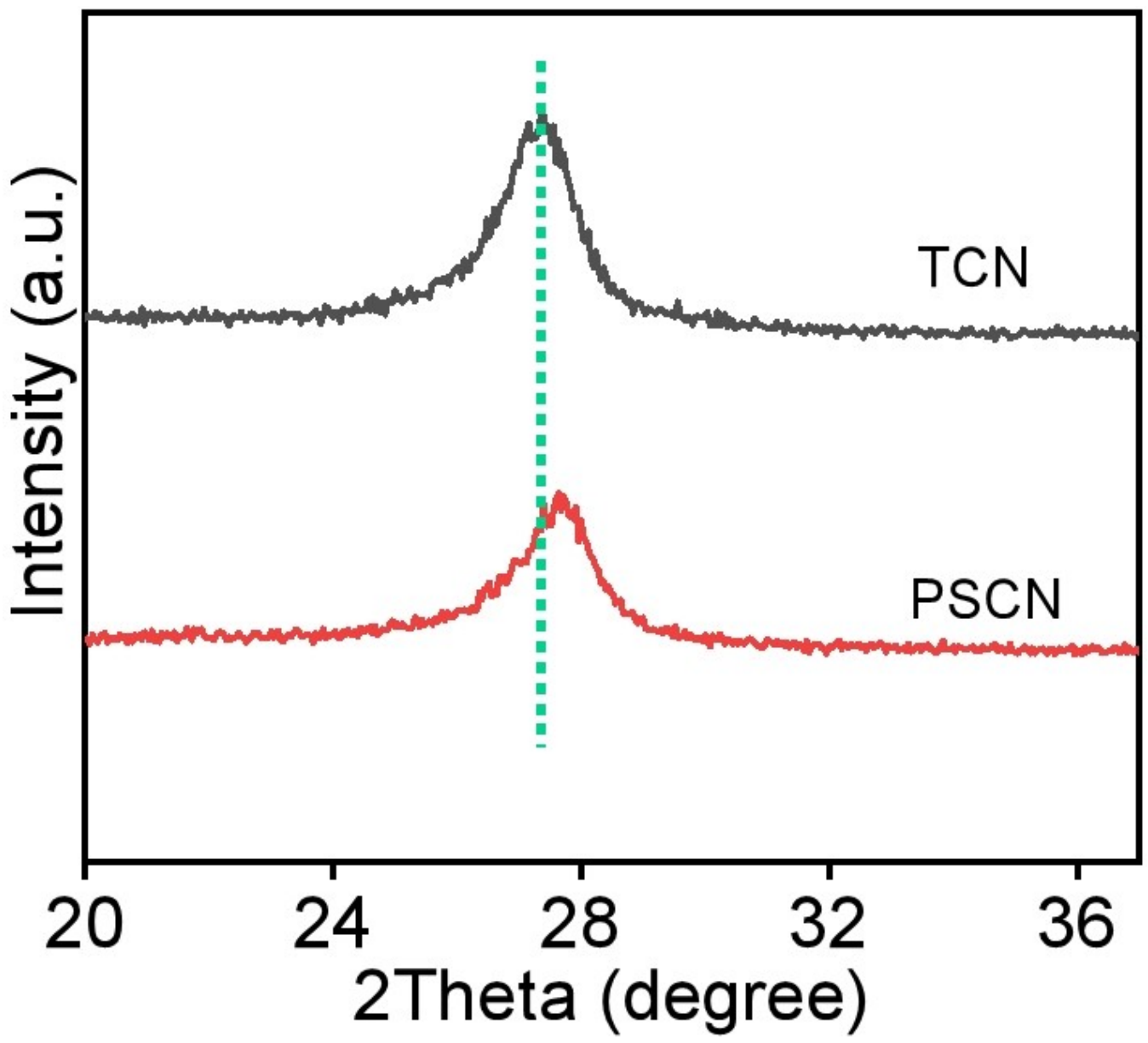

Fig. S1 Enlarged XRD pattern of TCN and PSCN.

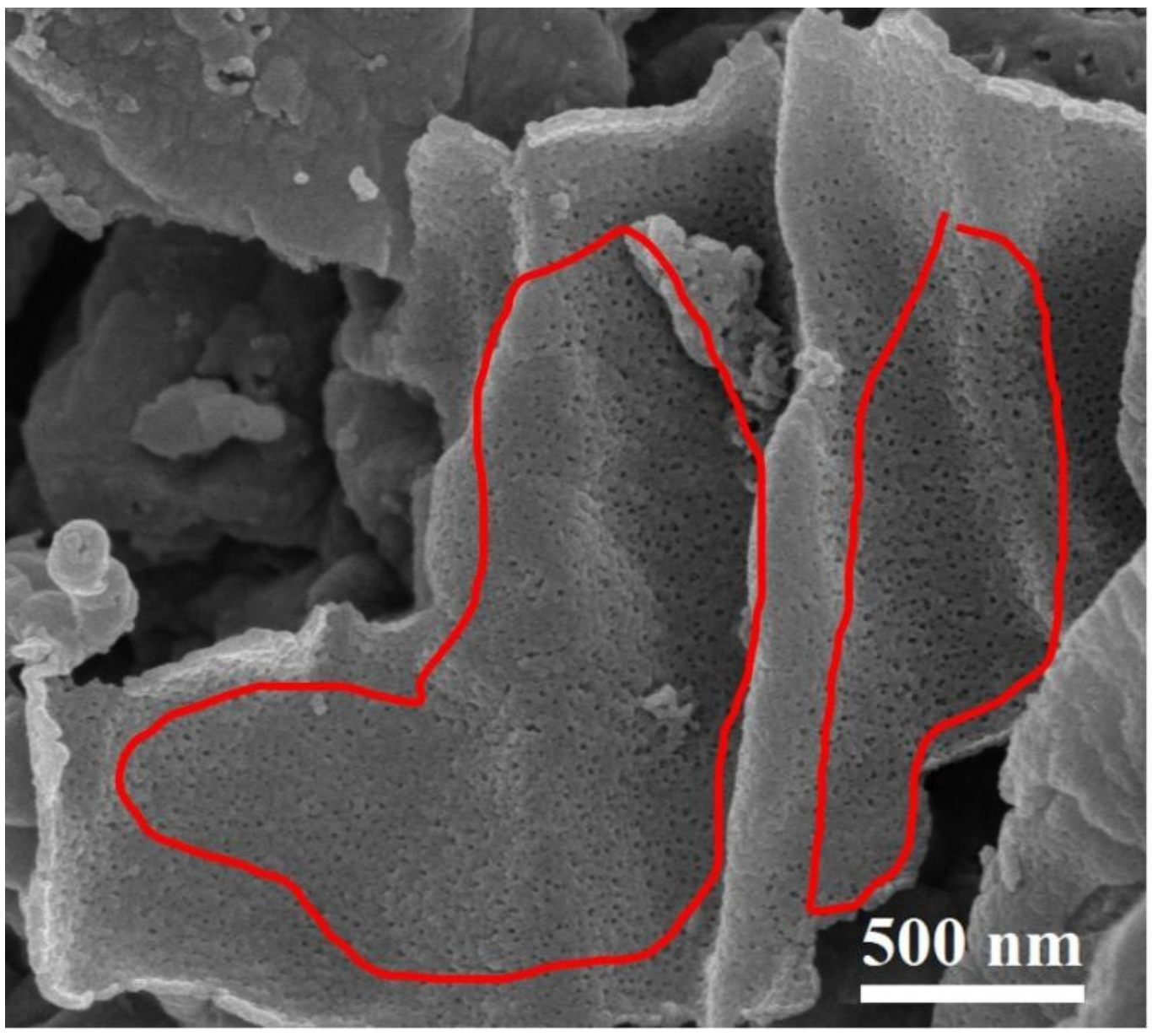

Fig. S2 SEM image of PSCN.

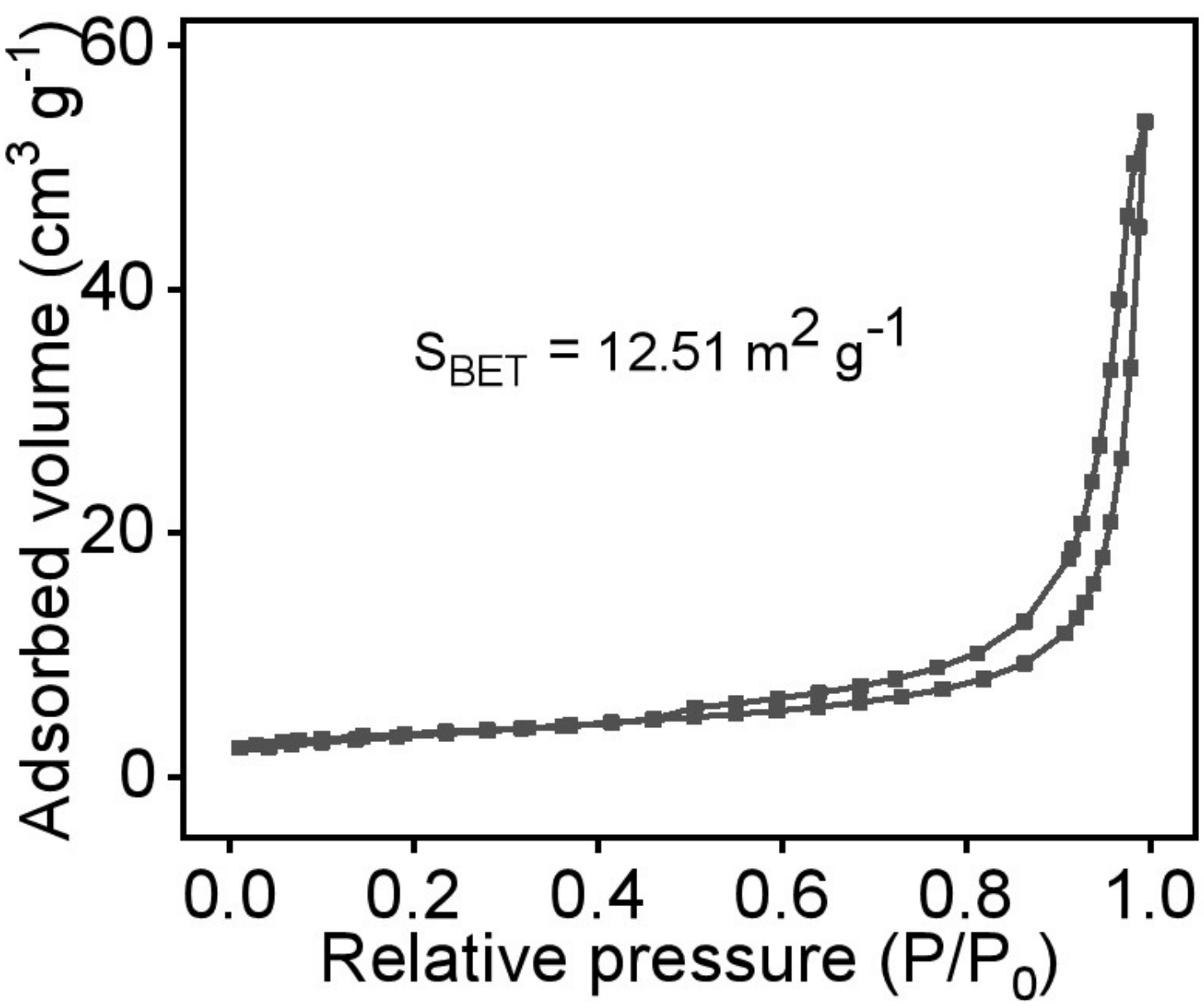

Fig. S3 N_2 adsorption–desorption isotherm curve of TCN sample.

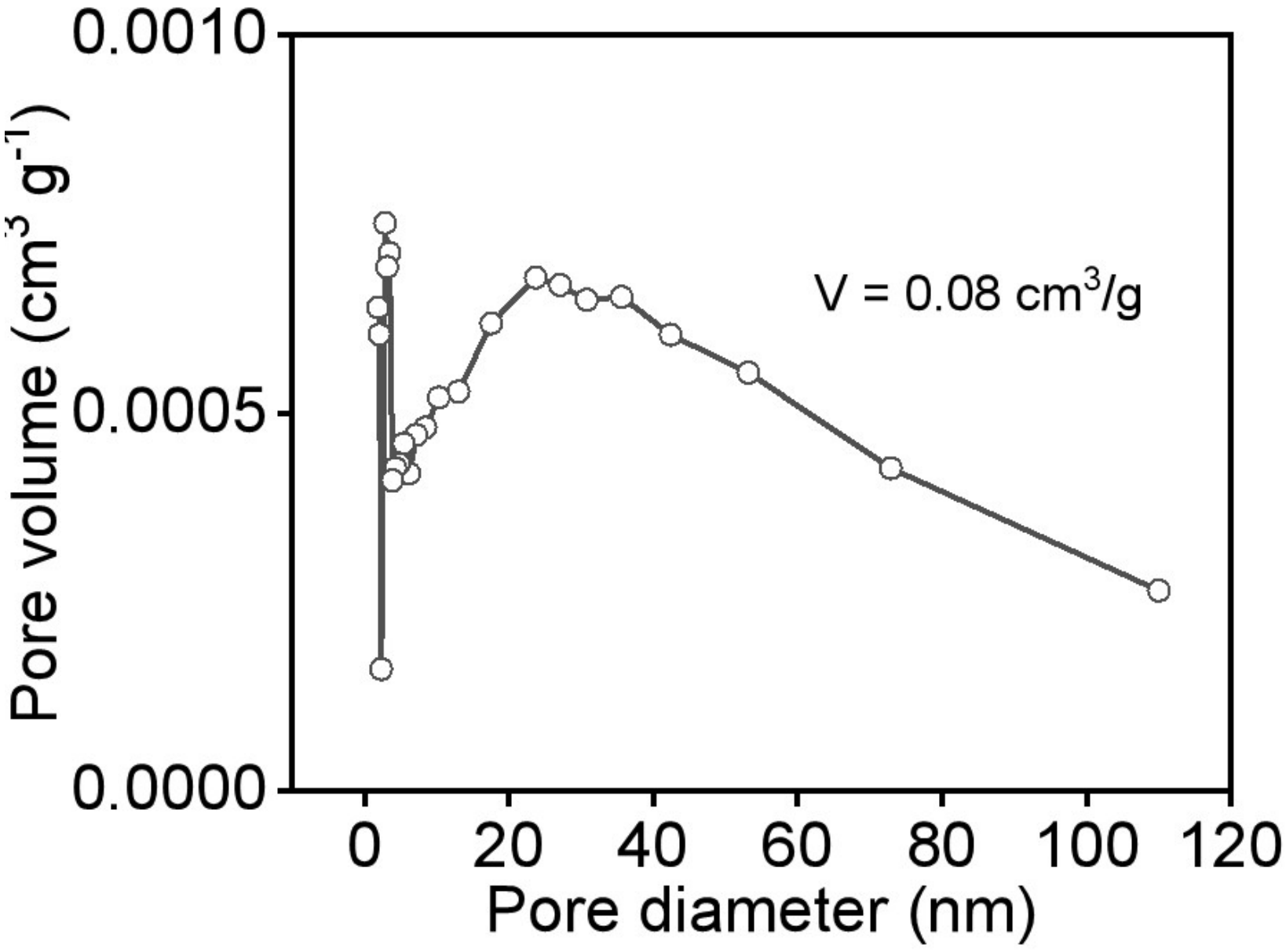

Fig. S4 Pore size distribution curve of TCN sample.

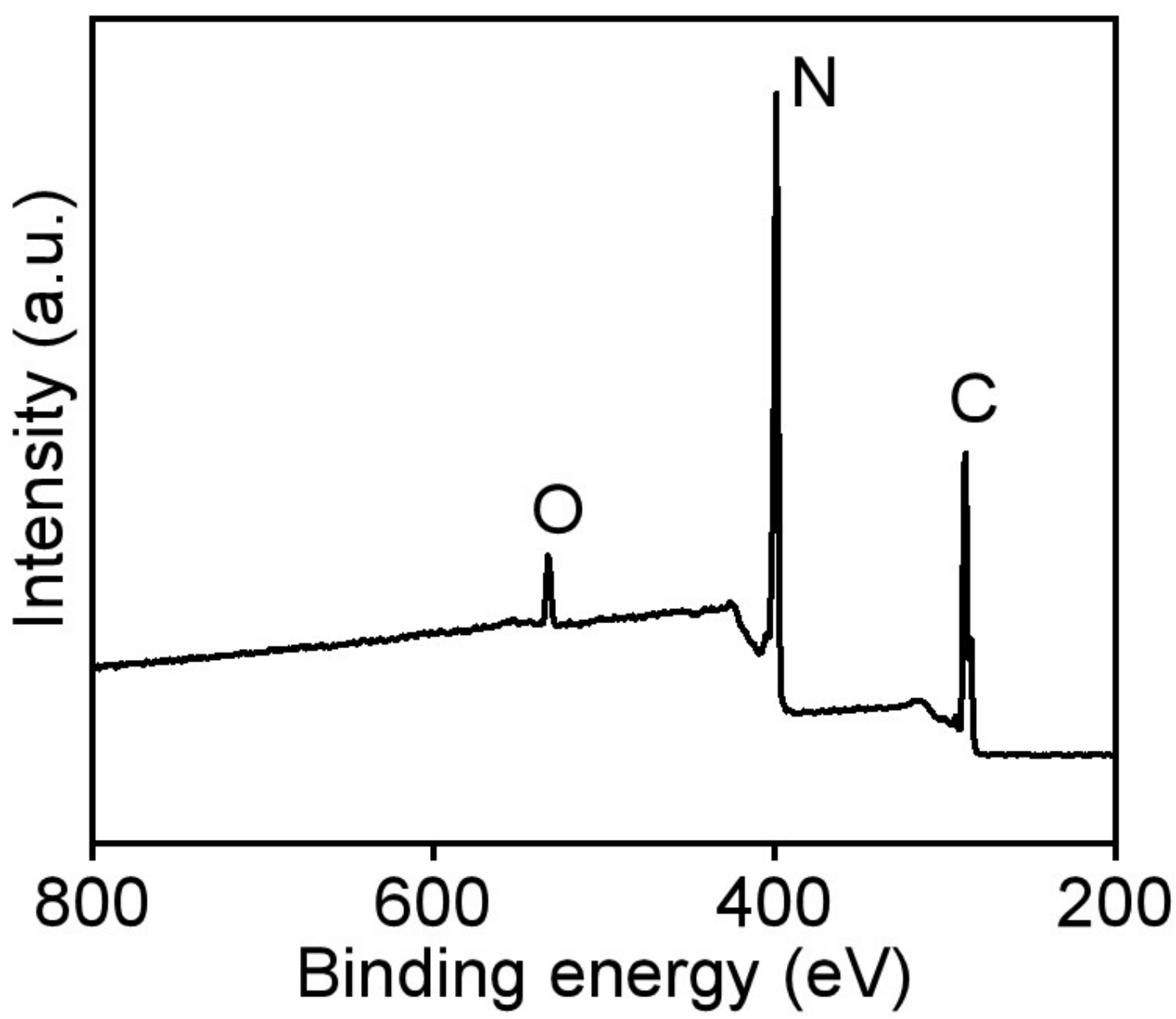

Fig. S5 Survey XPS spectra of as-fabricated TCN sample.

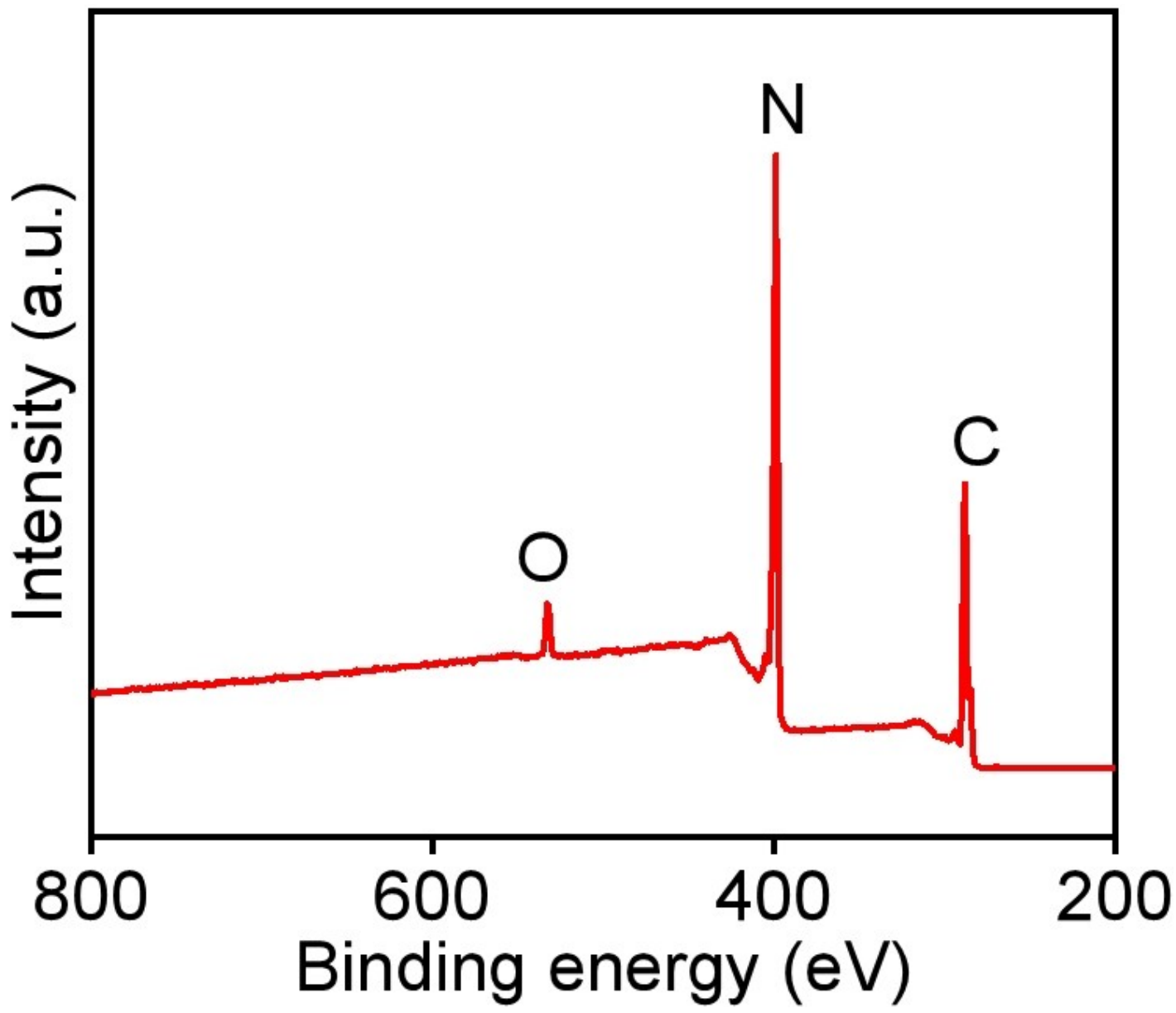

Fig. S6 Survey XPS spectra of as-fabricated PSCN sample.

Table S1 Relative ratios of C-N=C, N-$(C)_3$ and C-NH_2 determined by N 1s spectra.

Samples	C-N=C %	N-$(C)_3$ %	C-NH_2 %
TCN	77.28	12.33	10.39
PSCN	77.41	12.33	10.26

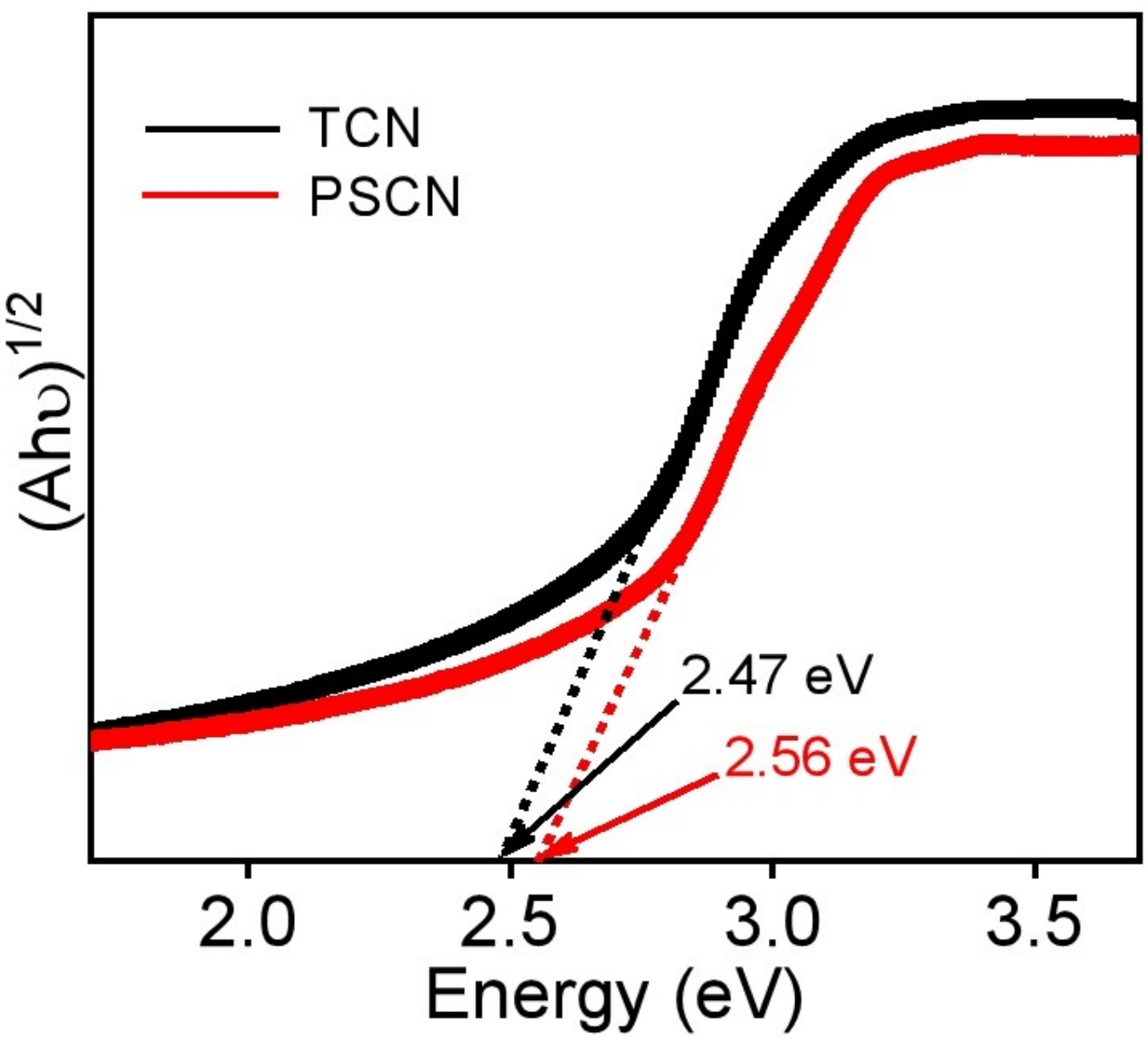

Fig. S7 Bandgap determined by Tauc plots of TCN and PSCN.

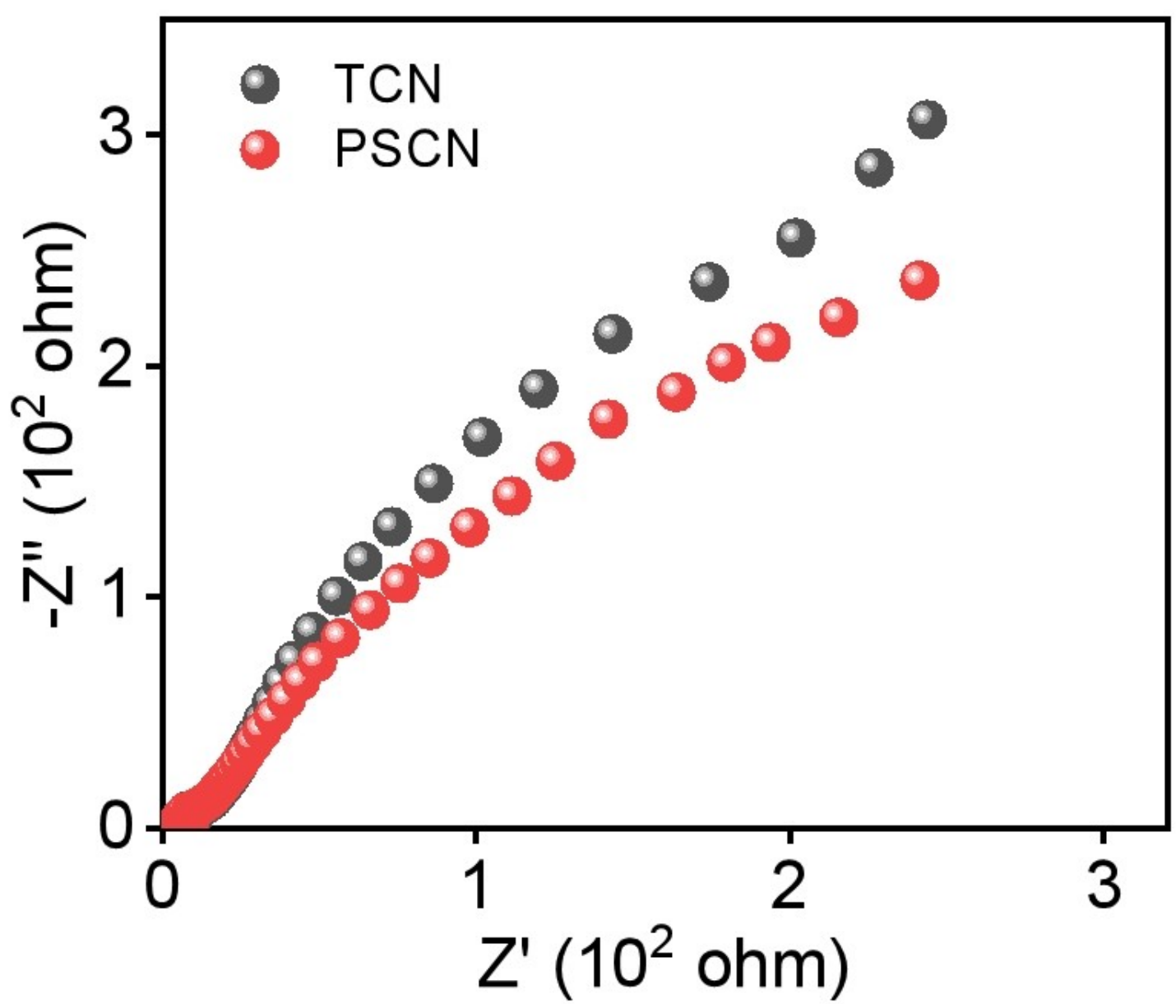

Fig. S8 EIS Nyquist plots of TCN and PSCN.

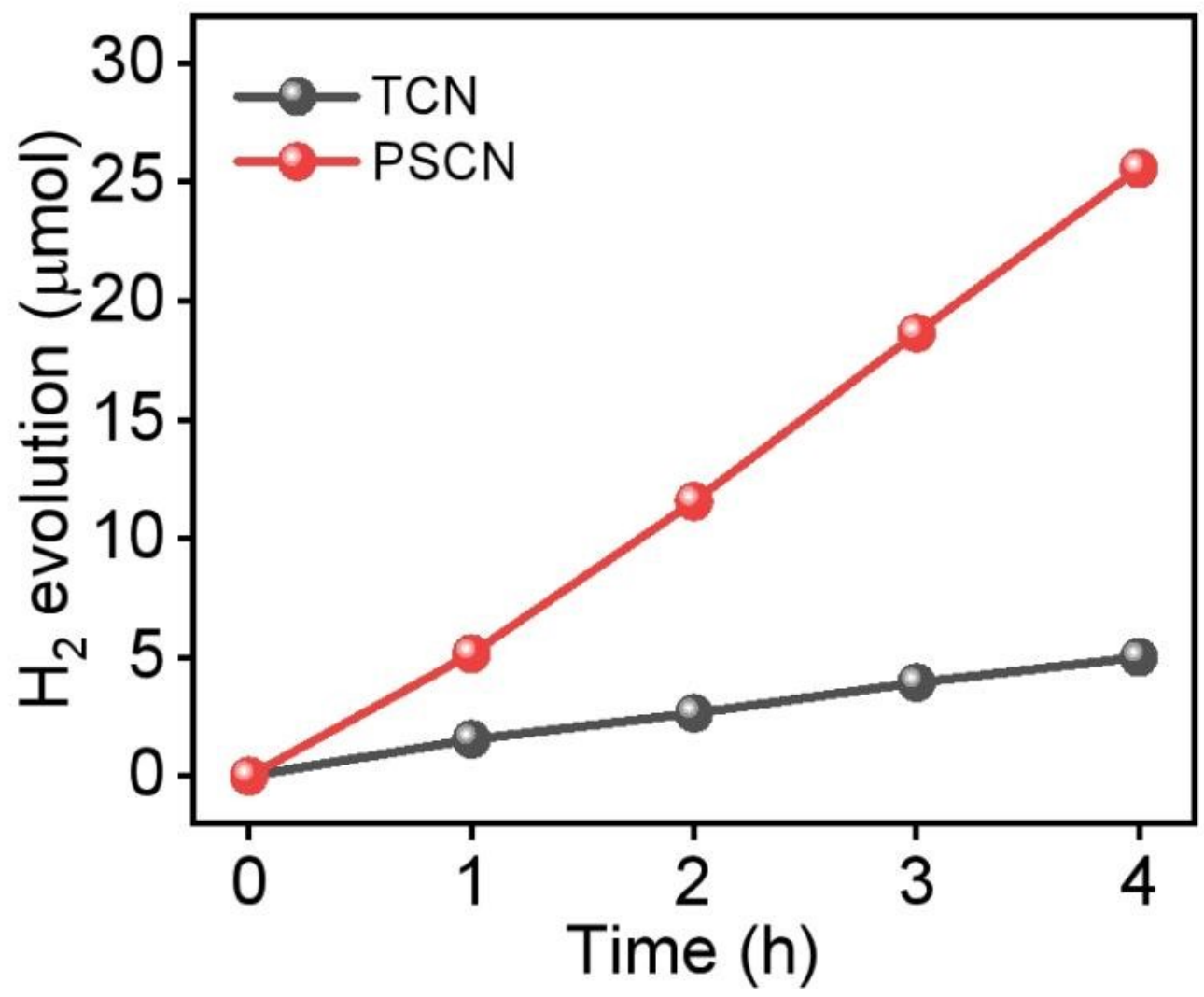

Fig. S9 Photocatalytic hydrogen evolution activity of TCN and PSCN.

Table S2. Performance comparison of photocatalysts based on carbon nitrides reported for H_2 evolution.

Photocatalyst	Light Source	Conditions	H_2 evolution rate ($\mu mol\ h^{-1}\ g^{-1}$)	Ref
FAT-1.0	300W Xe lamp (420 nm < λ < 710 nm)	3 wt% Pt 10 vol% TEOA	772	1
TSCN	300W Xe lamp (λ ≥ 420 nm)	3 wt% Pt 11% TEOA	630	2
IGCNSs	300W Xe lamp (λ ≥ 420 nm)	3 wt% Pt 10% TEOA	890	3
OH-CN3	300W Xe lamp (λ ≥ 420 nm)	1wt% Pt 10% lactic acid	310	4
CN aerogels	300W Xe lamp (λ ≥ 420 nm)	3 wt% Pt 10% TEOA	600	5
ACN	300W Xe lamp (λ ≥ 440 nm)	6 wt% Pt 10% TEOA	158	6
P-TCN	300W Xe lamp (λ ≥ 420 nm)	1 wt% Pt 20% methanol	670	7
CN16	300W Xe lamp (λ ≥ 420 nm)	0.5 wt% Pt 15 vol% TEOA	1002	8
Al-aa(0.32%)_PCN	300W Xe lamp (λ ≥ 420 nm)	3 wt% Pt 10 vol% TEOA	1200	9
PSCN	300W Xe lamp (λ ≥ 420 nm)	3 wt% Pt 10 vol% TEOA	1278	This work

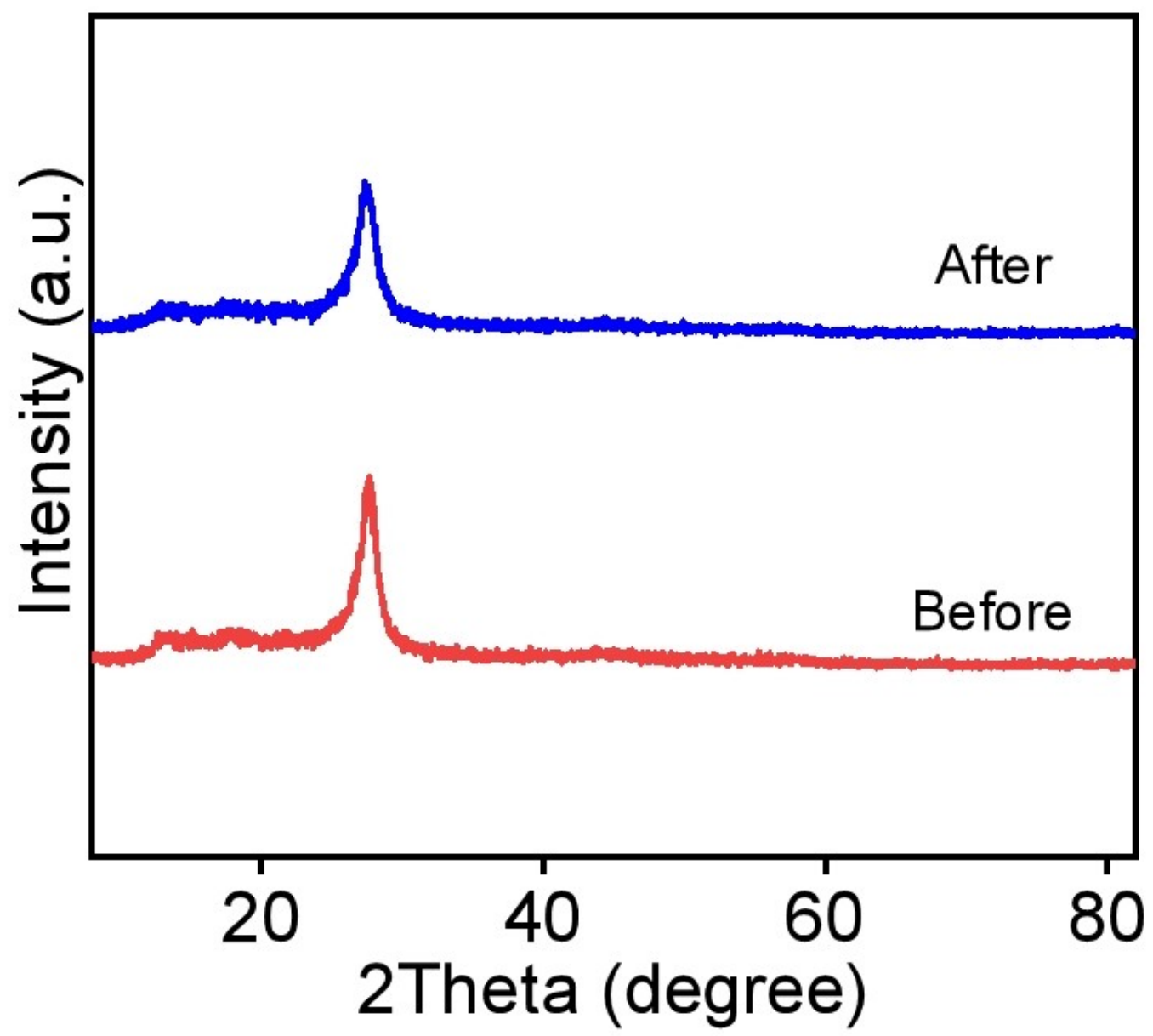

Fig. S10 The XRD patterns of PSCN photocatalyst before and after cycle test.

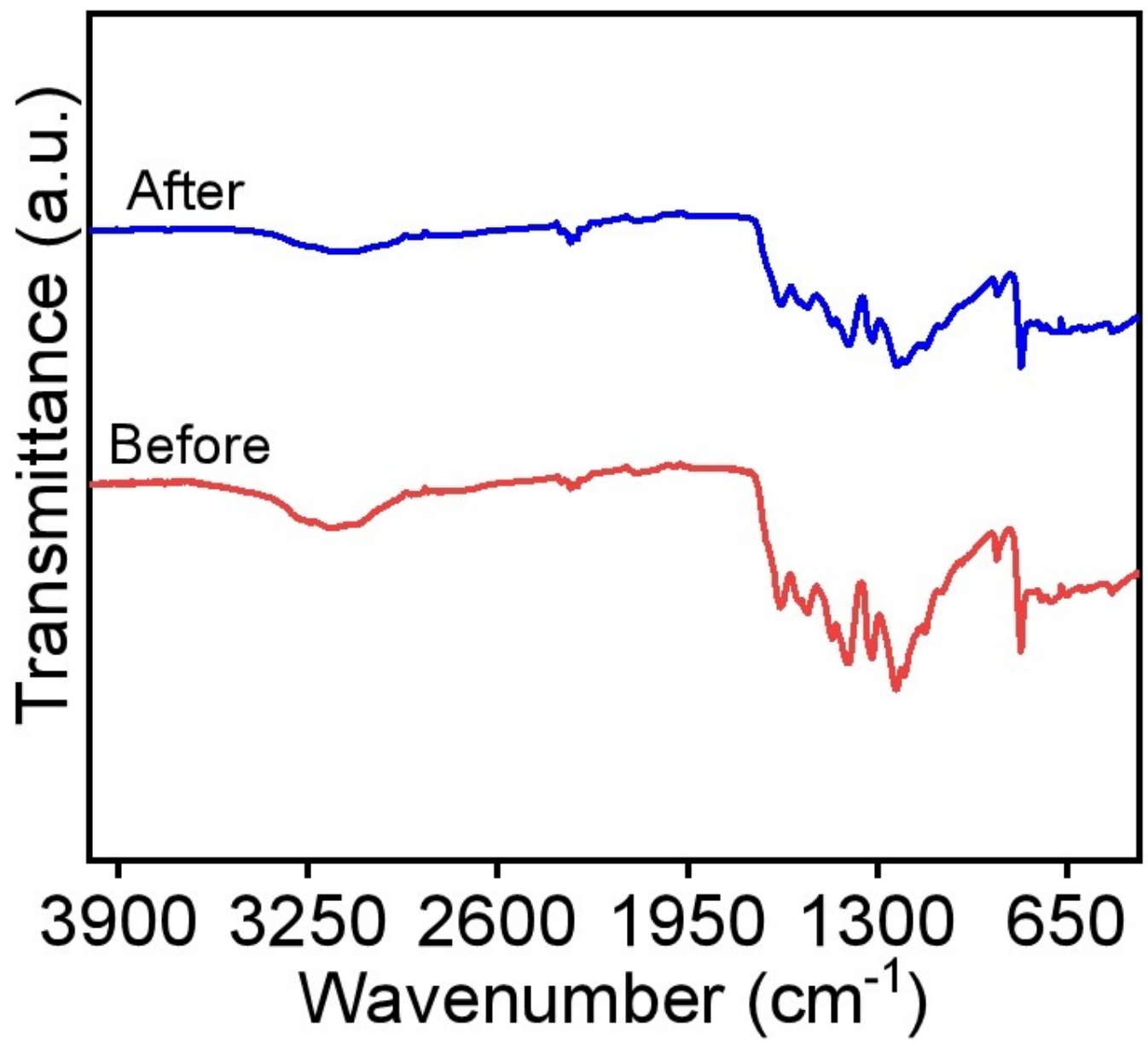

Fig. S11 The FTIR patterns of PSCN photocatalyst before and after cycle test.

References

1. Y. Wang, F. Silveri, M. K. Bayazit, Q. Ruan, Y. Li, J. Xie, C. R. A. Catlow and J. Tang, Bandgap engineering of organic semiconductors for highly efficient photocatalytic water splitting, Adv. Energy Mater., 2018, 8, 1801084.
2. D. Y. Z. Tong, Z. Li, Y. Nan, F. Ding, Y. Shen, Z. Jiang,, Thylakoid-inspired multi-shell g-C_3N_4 nanocapsules with enhanced visible-light harvesting and electron transfer properties for high-efficiency photocatalysis, ACS nano, 2017, 11, 1, 1103–1112.
3. Q. Han, C. Hu, F. Zhao, Z. Zhang, N. Chen and L. Qu, One-step preparation of iodine-doped graphitic carbon nitride nanosheets as efficient photocatalysts for visible light water splitting, J. Mater. Chem. A, 2015, 3, 4612-4619.
4. S. Yu, J. Li, Y. Zhang, M. Li, F. Dong, T. Zhang and H. Huang, Local spatial charge separation and proton activation induced by surface hydroxylation promoting photocatalytic hydrogen evolution of polymeric carbon nitride, Nano energy, 2018, 50, 383-392.
5. H. Ou, P. Yang, L. Lin, M. Anpo and X. Wang, Carbon Nitride Aerogels for the Photoredox Conversion of Water, Angew. Chem. Int. Ed., 2017, 56, 10905-10910.
6. Y. Kang, Y. Yang, L. C. Yin, X. Kang, G. Liu and H. M. Cheng, An amorphous carbon nitride photocatalyst with greatly extended visible-light-responsive range for photocatalytic hydrogen generation, Adv. Mater., 2015, 27, 4572-4577.
7. S. Guo, Z. Deng, M. Li, B. Jiang, C. Tian, Q. Pan and H. Fu, Phosphorus-doped carbon nitride tubes with a layered micro-nanostructure for enhanced visible-light photocatalytic hydrogen evolution, Angew. Chem. Int. Ed., 2015, 55, 1830-1834.
8. Y. Guo, J. Li, Y. Yuan, L. Li, M. Zhang, C. Zhou and Z. Lin, A Rapid Microwave-Assisted Thermolysis Route to Highly Crystalline Carbon Nitrides for Efficient Hydrogen Generation, Angew. Chem. Int. Ed., 2016, 55, 14693-14697.
9. C. H. Choi, L. Lin, S. Gim, S. Lee, H. Kim, X. Wang and W. Choi, Polymeric Carbon Nitride with Localized Aluminum Coordination Sites as a Durable and Efficient Photocatalyst for Visible Light Utilization, ACS Catal., 2018, 8, 4241-4256.
